# Tracheal intubation without neuromuscular block in children

**DOI:** 10.4103/0019-5049.60493

**Published:** 2010

**Authors:** Safiya I Shaikh, Vijayalaxmi P Bellagali

**Affiliations:** Department of Anesthesiology, Karnataka Institute of Medical Sciences, Hubli, Karnataka - 580 022, India

**Keywords:** Endotracheal intubation, Fentanyl, Propofol, suxamethonium

## Abstract

Endotracheal intubation has been performed during the administration of Propofol anaesthesia without neuromuscular blockade. In the study, we have assessed tracheal intubating conditions and haemodynamic responses in children aged 4 to12 years by using combination of either Fentanyl and Propofol; or Propofol and a neuromuscular blocker, suxamethonium. Intubating conditions were assessed on a 1-4 scale based on ease of laryngoscopy, position of vocal cords, degree of coughing and jaw relaxation. Tracheal intubation was successful in 95% of patients receiving Fentanyl-Propofol and 100% of patients receiving Propofol-suxamethonium. Fentanyl-Propofol provided better haemodynamic stability than Propofol-suxamethonium. We conclude that Propofol-Fentanyl combination could be a useful alternative technique for tracheal intubation when neuromuscular blocking drugs are contraindicated or need to be avoided.

## INTRODUCTION

Endotracheal intubation is frequently facilitated by administration of a depolarizing muscle relaxant such as suxamethonium during induction of anaesthesia with short-acting hypnotic drugs. However, suxamethonium administration may be associated with side effects such as postoperative myalgia, prolonged paralysis, increase in intraocular pressure and hyperkalaemia.[1]

Routine use of suxamethonium for tracheal intubation in children is being criticized following some reports of cardiac arrest and death in young children.[2] Even the use of nondepolarizing relaxants may be associated with undesirable effects such as prolonged neuromuscular blockade, the need to reverse neuromuscular blockade or the inability to reverse the paralysis quickly if airway management via mask or tracheal intubation is not possible. For these reasons, a method of providing good intubating conditions rapidly without using muscle relaxants has been sought by a number of investigators[2]

Propofol has been reported to possess some characteristics that provide adequate conditions for intubation in combination with Fentanyl[3,4] or alfentanil[5–8] or remifentanil.[9,10]

The purpose of the present study was to assess intubating conditions and haemodynamic responses in children after induction of anaesthesia using Fentanyl-Propofol and to compare the results with those obtained with a Propofol-suxamethonium induction sequence.

## METHODS

After institutional ethical clearance, 80 children aged 4 to 12 years, belonging to American Society of Anaesthesiologists (ASA) grade I and II, were included in this study. The children posted to undergo various elective surgical procedures, for which endotracheal anaesthesia was planned, were selected for study. Children with suspected difficult intubation, having history of allergy to any of the study drugs, undergoing ophthalmic and neurosurgical operations were excluded from the study.

Informed and written parental consent was obtained. Patients were allotted to one of the following groups based on computer-based randomization: Group F- to receive Inj. Fentanyl 4 *μ*g/kg + Inj. Propofol 3 mg/kg and

Group S- to receive Inj. Propofol 3 mg/kg + Inj. suxamethonium 1 mg/kg.

All the patients were pre-medicated with Inj. Midazolam 0.05 mg/kg and atropine 0.01 mg/kg I.V., 10 minutes prior to induction.

Group F (study group)- Inj. Fentanyl 4*μ*g/kg was given I.V. over 30 seconds. Five minutes later, the children received Propofol 3 mg/kg over a period of 30 seconds (Lignocaine 0.2 mg/kg was added to Propofol solution to abolish pain on injection). Laryngoscopy and intubation were attempted 60 seconds after induction of anaesthesia in both the groups. Additional bolus of 1 mg/kg of Propofol was given if laryngoscopy was not possible due to muscle spasm, coughing or excessive movements. In those patients where intubation was impossible after two attempts due to any cause, suxamethonium 1 mg/kg was injected and intubation completed.

In Group S (control group), anaesthesia was induced by Inj. Propofol 3 mg/kg followed by Inj. suxamethonium 1 mg/kg; endotracheal intubation was performed 60 seconds later.

Laryngoscopy and intubation were done in all the patients by a senior consultant anaesthesiologist. The quality of intubation was graded by the consultant using the scoring system devised by Helbo-Hansen Raulo and Trap-Anderson[11] [Table 1].

**Table 1:** Scoring criteria for intubating conditions

	1	2	3	4
Laryngoscopy	Easy	Fair	Difficult	Impossible
Vocal cords	Open	Moving	Closing	Closed
Coughing	None	Slight	Moderate	Severe
Jaw relaxation	Complete	Slight	Stiff	Rigid

During laryngoscopy and intubation, the intubating anaesthesiologist assessed each patient for four variables [Table 1]:

- Ease of laryngoscopy
- Position of vocal cords
- Degree of coughing
- Jaw relaxation

The observed conditions with respect to each of the above were allocated scores of 1 to 4. A score of 3-4 was considered excellent; 5-8, good; 9-12, poor; and 13-16, bad. Excellent and good scores were considered as clinically acceptable, and fair and poor scores were considered as clinically unacceptable.

Measurements of heart rate, systolic arterial pressure and arterial O_2_ saturation were noted at different time intervals (pre-induction, post-induction, post-intubation at 0, 1, 3 and 5 minutes). Measurements at 1 minute after injection of atropine were taken as baseline values.

Balanced anaesthesia was maintained subsequently as necessary for each case.

### Statistical analysis

The results were expressed as mean with standard error of mean as index of dispersion. Blood pressure, pulse rate and arterial O_2_ saturation were compared with baseline values using paired *t* test. Comparison of variables obtained with Propofol-Fentanyl was done with those obtained with Propofol-suxamethonium using Fisher exact test. *P*<0.05 was regarded as statistically significant, *P*<0.001 was taken as highly significant and *P*>0.05 was regarded as not significant. For sample size calculation, we considered excellent and good conditions as acceptable whereas fair and poor as non-acceptable. Sample size was decided in consultation with the statistician: Thirty was the smallest number in each group, where any results could be statistically significant (with power of 80%). Hence sample size of 40 patients was selected for both the groups. The Fisher exact test was used to compare the intubation scores.

## RESULTS

All the patient parameters and the results from the two groups (group F and group S) were entered in the pre-designed study pro forma sheet, intubating conditions were scored and haemodynamic parameters were noted.

There was no significant difference in demographic data for both the groups [Table 2].

**Table 2:** Patient data (mean±SD)

	Group F	Group S
Number of patients	40	40
Age (years)	7.875±2.821	8.83±2.38
Weight (kg)	19.7±6.13	21±5.5
Male/Female	28/12	19/21

F: Fentanyl, S: Suxamethonium, SD: Standard deviation

The scores observed in each group based on the criteria used to assess ease of intubation [Table 1] are shown in Table 3. *Excellent* intubating conditions (intubation score, 3-4) were achieved in 14 (35%) out of 40 patients in group F and 36 (90%) out of 40 patients in group S. *Good* intubating conditions (intubation score, 5-8) were achieved in 24 (60%) patients in group F and 4 (10%) patients in group S. In patients with a score of 1 to 2, laryngoscopy was easy, the vocal cords were open, cough was neither observed or was too minimal to impede the passage of the tracheal tube [Tables 3 and 4].

**Table 3:** Comparison of scoring criteria

	Laryngoscopy				VC position				Coughing				Jaw mobility			
				
	1	2	3	4	1	2	3	4	1	2	3	4	1	2	3	4
Group F	35	3	2	0	30	8	2	0	14	24	1	1	32	6	2	0
Group S	36	4	0	0	36	4	0	0	40	0	0	0	38	2	0	0

F: Fentanyl, S: suxamethonium

**Table 4:** Scoring conditions for tracheal intubation

Group	Score3-4 (Excellent %)	Score 5-8 (Good %)	Score 9 -12 (Fair %)	Score 13-16 (Poor %)
F (*n* = 40)	14 (35)	24 (60)	1 (2.5)	1 (2.5)
S (*n* = 40)	36 (90)	4 (10)	0	0

F: Fentanyl, S: Suxamethonium.

*Fair* intubating conditions (intubation score, 9-12) were observed in 1 (2.5%) out of 40 patients in group F as compared to 0 in group S [Table 4]. This patient was having a score of 12 with difficult laryngoscopy, stiff jaw, vocal cord closing and moderate cough in response to intubation. Poor intubating conditions (intubation score, 13-16) were observed in 1 (2.5%) patient in group F and in no patient in group S. This patient had stiff jaw, difficult laryngoscopy, closing vocal cords and *severe* cough in response to intubation (intubation score, 13). For both these patients, belonging to group F, additional bolus dose of 1 mg/kg Propofol was administered, and a second attempt of intubation was made. Since this could not facilitate intubation, suxamethonium 1 mg/kg was administered and intubation was completed.

### Overall intubating conditions

*Acceptable intubating conditions* (i.e., excellent and good) were observed in 38 (95%) out of 40 patients in group F, whereas all (100%) patients in group S had excellent intubating conditions (not statistically significant).

*Unacceptable intubating conditions* were observed in 2 (5%) out of 40 patients in group F and none in group S; this was not statistically significant [Table 5]. *In all unacceptable intubating conditions* (fair and poor) were present in 2 (5%) out of 40 patients in group F and no patient in group S; this was not statistically significant.

**Table 5:** Intubating conditions in the two groups

Intubating conditions	Group I F	Group II S	*P* value
Acceptable (Excellent +good)	38/40 (95)	40/40 (100)	NS
Not acceptable (Fair + Poor)	2/40 (5)	0	NS

NS: not significant (P>0.05), F: fentanyl, S: suxamethonium, Figures in parenthesis are in percentage

### Haemodynamic changes during intubation

**Figure 1:**
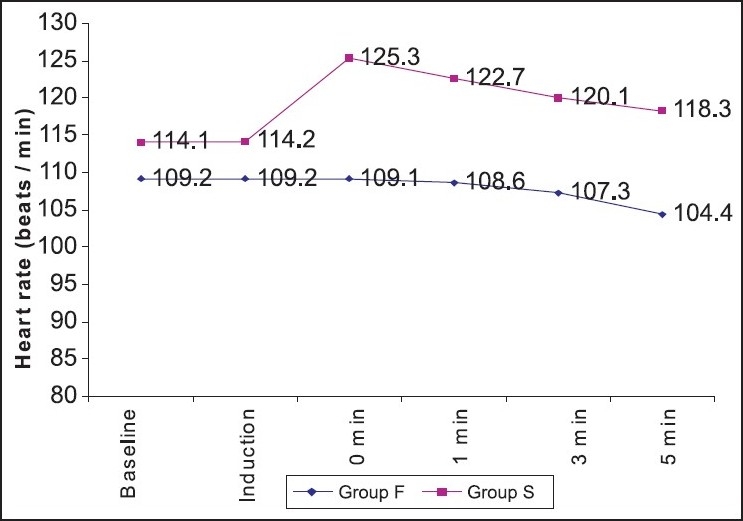
Comparison of heart rate

**Figure 2:**
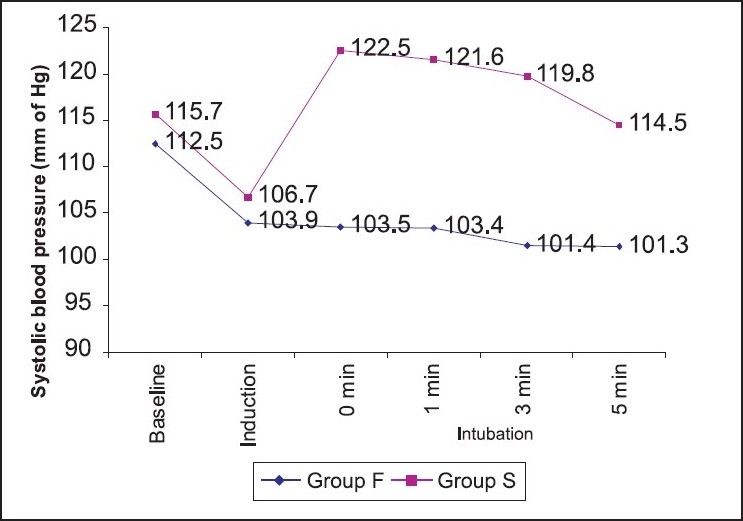
Comparison of systolic blood pressure

The mean basal heart rate was 109.2±11.7/min in group F and 114.1±11.4/min in group S, both of which were not statistically significant (*P*>0.05) [Figure 1]. There was significant decrease in heart rate in group F after intubation at 0, 1, 3 and 5 minutes (*P*<0.001), whereas group S showed significant increase in heart rate after intubation at 0, 1, 3 and 5 minutes (*P*<0.001) [Figures 1 and 2].

The pre-induction systolic blood pressure was 112.5±6.39 mm Hg in group F and 115.7±9.03 mm Hg in group S, respectively, both of which were not statistically significant. The systolic blood pressure decreased significantly after intubation at 0, 1, 3 and 5 minutes in group F (*P*<0.001), whereas group S showed significant increase in systolic blood pressure at 0, 1, 3 and 5 minutes (*P*<0.001) [Figure 2].

There was no significant change in arterial oxygen saturation in group F compared to groups S during the study period.

## DISCUSSION

Tracheal intubation without the use of neuromuscular blocking drugs is a technique which has been widely studied and practiced following the work of MC Keating, Bali and Dundee. The study showed that conditions for laryngoscopy were superior after induction of anaesthesia with Propofol rather than thiopentone.[12] Propofol decreases laryngotracheal reactivity and muscle tone and thus allows ease in intubation,[13] but the intubating conditions are not optimal.[5,14] Increasing the depth of anaesthesia by administering supplementary increments of induction agent or opioids or lignocaine improves intubating conditions.[7,15] Administration of Fentanyl suppresses the haemodynamic response to endotracheal intubation.[16] The observation that Propofol causes greater suppression of laryngeal reflexes has renewed interest in the use of relaxant-free techniques of tracheal intubation. Intubating conditions attained using Propofol alone, however, are far from ideal and have been considered adequate in only 38% to 60% of patients.[14,17,18] Addition of opioids improved intubation conditions.[3,12,18–24] Batra *et al.*[24] concluded that remifentanil (3 *μ*g/kg) administered before Propofol 3 mg/kg provides acceptable tracheal intubating conditions in children and completely inhibits the increase in heart rate associated with intubation.

Based on the respective findings of Gupta and others,[19] Andel *et al.,*[25] and Ko *et al.,*[26] a Propofol-Fentanyl technique was used for the current study. Gupta and others in their study on evaluation of different doses of Propofol with prior administration (3 minutes before) of 3 *μ*g/kg of Fentanyl in children in the age group of 3 to 10 years found a dose of Propofol of 3.5 mg/kg to be effective in producing acceptable intubating conditions. Doses of 3 to 3.5 mg/kg of Propofol produced good attenuation of haemodynamic responses to intubation. Andel and others determined the required Propofol dose in combination with Fentanyl allowing reliably successful tracheal intubation without neuromuscular blocking agents in all patients. According to their finding, a median Propofol dose of 2.7 mg/kg is needed. Regarding the use of Fentanyl in this context, Ko, *et al*[26] reported that in terms of blunting the haemodynamic response to laryngoscopy and tracheal intubation, it was more effective to administer the bolus dose of Fentanyl 5 minutes before intubation.

Based on the above studies, in our study 4 μg/kg Fentanyl was given 5 minutes before intubation, and induction dose of Propofol 3 mg/kg was used. An additional advantage is the ability to maintain spontaneous breathing in case of intubation failure as a result of airway pathology. Lignocaine in the dose of 0.2 mg/kg body weight was mixed with Propofol to avoid pain on injection. Lignocaine has been used in many studies in the past as adjuvant. It attenuates the intraocular pressure response to rapid tracheal intubation in children. It has been shown to attenuate the pressure response to laryngoscopy and tracheal intubation, but timings of administration of doses are important.

The present study was carried out in children to assess tracheal intubating conditions and haemodynamic changes after induction of anaesthesia by using Fentanyl-Propofol without the use of neuromuscular blocking drugs. This was compared with the standard technique of using Propofol-suxamethonium. Out of 80 patients, 40 received Fentanyl-Propofol and 40 received Propofol- suxamethonium.

Our results showed that tracheal intubation was successful in 95% of children receiving Fentanyl-Propofol and 100% of patients receiving Propofol-suxamethonium. Only 2 out of 40 patients had *unacceptable intubating conditions* in the Fentanyl-Propofol group, requiring administration of suxamethonium for intubation. The overall scores for ease of laryngoscopy, the position of vocal cords, relaxed jaws and absence of coughing were however better in the Propofol-suxamethonium group. Olmos, Stribel and colleagues[3] were successful in intubating more than 95% of adult patients given Fentanyl and Propofol. They stated that combination of Fentanyl, thiopentone and succinylcholine results in no better intubating conditions than Fentanyl plus Propofol. Gupta,[19] Tahira[12] and de Fatima[20] also concluded that Propofol-Fentanyl provided adequate tracheal intubating conditions without significant haemodynamic changes. On the contrary, Uma Srivastava *et al.,*[3] Mencke Thomas *et al.*[27] and Samar *et al.*[28] have achieved lower success rate despite augmentation of Propofol with Fentanyl. Tsuda *et al.*[17] also found that low-dose Fentanyl in the presence of Propofol provided poor intubating conditions.

Regarding the haemodynamic effects of the different combinations for anaesthetic induction and intubation, Mark *et al.*[18] conducted the study in infants and showed that Propofol-Remifentanil provides clinically acceptable intubating conditions and stable haemodynamics. Our results showed that after intubation, heart rate decreased significantly in patients who received Fentanyl and Propofol, whereas heart rate was increased in patients given Propofol-suxamethonium. This has been observed by several other investigators.[4,5,9] Our results showed that systolic blood pressure was decreased in Propofol-Fentanyl group after intubation, whereas it increased in the suxamethonium group. The fall in systolic blood pressure from the pre-induction value was highly significant in the Propofol-Fentanyl group. The fall in systolic blood pressure is comparable to that in studies by Uma Srivastava *et al.,*[2] Tahira Shah[12] and Billard *et al.*[29] Randall and others[30] concluded that low-dose Fentanyl reduces some aspects of stress response to rapid-sequence induction of anaesthesia. Dahlgren and Messeter[31] have also shown that low-dose Fentanyl before intubation effectively blunts the haemodynamic response to intubation. Gupta and others[19] found that a dose of 3 mg/kg of Propofol with a Fentanyl dose of 3*μ*g/kg was the best combination to reduce intubation responses, without greater falls in mean arterial pressure and heart rate. The administration of Propofol in a dose of 2-2.5 mg/kg can lower mean blood pressure by 25% to 40%. This drop is secondary to both the vasodilator and the myocardial depressant effects of Propofol. In view of the drop in mean arterial pressure, this technique of tracheal intubation without muscle relaxants may not be appropriate for elderly patients and in patients with cardiovascular or cerebrovascular disease.[27]

Muscle rigidity following opiate administration has been studied in human volunteers, and previous reports show that rigidity occurs in 80% of patients when 175 *μ*g/kg of alfentanil is administered and in 50% of patients when 15μg/kg of Fentanyl was used[30] Muscle rigidity was not observed during our study. The absence of muscle rigidity in our study can be attributed to the much lower dosage of narcotic used and also to our slow injection rate of narcotics, since there is evidence that the incidence and severity of opiate-induced rigidity are not only dependent on the dosage but also on the rate of administration.[1]

Our study had the limitation of lack of double blinding; the same study with a double blinding is in progress. If confirmed in further trials, the findings may lead to modification of the scoring system presently used.

## CONCLUSION

The present study was undertaken to highlight the benefits of avoiding suxamethonium, using only the opioid-Propofol technique for routine intubation in paediatric age groups. We conclude that in pre-medicated healthy children, tracheal intubation may be accomplished using a combination of Fentanyl (4 *μ*g/kg) and Propofol (3 mg/kg). The simultaneous administration of muscle relaxant may not be necessary to ensure acceptable jaw mobility, easy laryngoscopy and vocal cord exposure. This method represents a useful alternative technique for tracheal intubation when neuromuscular blocking drugs are contraindicated or should be avoided.

## References

[1] Scheller MS, Zornow MH, Saidman LJ (1992). Tracheal intubation without the use of muscle relaxants. A technique using Propofol and varying dose of alfentanil. Anesth Analg.

[2] Srivastava U, Kumar A, Gandhi NK, Saxena S, Agarwal S (2001). Comparison of Propofol and Fentanyl with thiopentone and suxamethanium for tracheal intubation in children. Indian J Anaesth.

[3] Striebel HW, Hölzl M, Rieger A, Brummer G (1995). Endotracheal intubation with Propofol and Fentanyl. Anaesthesist.

[4] Olmos M, Ubierna B, Ruano C (1993). Intubation with Propofol without neuromuscular blockade. Effect of premedication of Fentanyl and lidocaine. Rev Esp Anestesiol Reanim.

[5] Beck GN, Masterson GR, Richards J, Bunting P (1993). Comparison of intubation following Propofol and alfentanil with intubation following thiopentone and suxamethonium. Anaesthesia.

[6] McConaghy P, Bunting HE (1994). Assessment of intubating conditions in children after induction with Propofol and varying doses of alfentanil. Br J Anaesth.

[7] Steyn MP, Quinn AM, Gillespie JA, Miller DC, Best CJ, Morton NS (1994). Tracheal intubation without neuromuscular block in children. Br J Anaesth.

[8] Collins L, Prentice J, Vaghadia H (2000). Tracheal intubation of outpatients with and without muscle relaxants. Can J Anaesth.

[9] Alexander R, Booth J, Olufolabi AJ, El-Moalem HE, Glass PS (1999). Comparison of remifentanil with alfentanil or suxamethonium following Propofol anesthesia for tracheal intubation. Anaesthesia.

[10] Klemola UM, Mennander S, Saarnivaara L (2000). Tracheal intubation without the use of muscle relaxants; remifentanil or alfentanil in combination with Propofol. Acta Anaesthesiol Scand.

[11] Helbo-Hansen S, Ravlo O, Trap-Andersen S (1988). The influence of alfentanil on the intubating conditions after priming with vecoronium. Acta Anaesthesiol Scand.

[12] Shah TS (2004). Tracheal intubation without neuromuscular block in children. J Postgrad Med.

[13] Coghlan SF, McDonald PF, Csepregi G (1993). Use of alfentanil with Propofol for nasotracheal intubation without neuromuscular block. Br J Anaesth.

[14] Saarnivaara L, Klemola UM (1991). Injection pain, intubating conditions and cardiovascular changes following induction of anesthesia with Propofol alone or in combination with alfentanil. Acta Anaesthesiol Scand.

[15] Davidson JA, Gillespie JA (1993). Tracheal intubation after induction of anesthesia with Propofol and alfentanil and I.V. lignocaine. Br J Anaesth.

[16] Adachi YU, Satomoto M, Higuchi H, Watanabe K (2002). Fentanyl attenuates the haemodynamic response to endotracheal intubation more than the response to laryngoscopy. Anesth Analg.

[17] Tsuda A, Yasumoto S, Akazawa T, Nakahara T (2001). Tracheal intubation without muscle relaxants using Propofol and varying doses of Fentanyl. Masui.

[18] Mark WC, Jason it, Juliana MT (2005). Dose response of remifentanil for tracheal intubation in infants. Anaesth Analg.

[19] Gupta A, Kaur R, Malhotra R, Kale S (2006). Comparative evaluation of different doses of Propofol preceded by Fentanyl on intubating conditions and pressor response during tracheal intubation without muscle relaxants. Paediatr Anaesth.

[20] de Fátima de Assunção Braga A, Da Silva Braga FS, Potério GM, Filier PR, Cremonesi E (2001). The effect of different doses of Propofol on tracheal intubating conditions without muscle relaxant in children. Eur J Anaesthesiol.

[21] Klemola UM, Hiller A (2004). Tracheal intubation after induction of anesthesia in children with Propofol-remifentanyl or Propofol-rocuronium. Can J Anaesth.

[22] Taha S, Siddik-Sayyid S, Alameddine M, Wakim C, Dahabra C, Moussa A (2005). Propofol is superior to thiopental for intubation without muscle relaxants. Can J Anaesth.

[23] Sussan SM, Farhood T (2006). Comparison of Propofol – remifentanil with thiopentone- remifentanil for tracheal intubation without using muscle-relaxants, a double blind randomized and clinical trial study. Int J Pharm.

[24] Batra YK, Al Qattan AR, Ali SS, Qureshi MI, Kuriakose D, Migahed A (2004). Assessment of tracheal intubating conditions in children using Propofol and remifentanil. Paediatr Anaesth.

[25] Andel H, Klune G, Andel D, Felfernig M, Donner A, Schramm W (2000). Propofol without muscle relaxants for conventional or fiberoptic nasotracheal intubation: A dose-finding study. Anesth Analg.

[26] Ko SH, Kim DC, Han YJ, Song HS (1998). Small dose Fentanyl: optimal time of injection for blunting the circulatory response to tracheal intubation. Anesth Analg.

[27] Mencke T, Echternach M, Kleinschmidt S, Lux P, Barth V, Plinkert PK (2003). Laryngeal morbidity and quality of tracheal intubation; a randomised controlled trial. Anesthesiology.

[28] Jabbour-Khoury SI, Dabbous AS, Rizk LB, Abou Jalad NM, Bartelmaos TE, El-Khatib MF (2003). A combination of alfentanil – Lidocaine – Propofol in the absence of muscle relaxants. Can J Anaesth.

[29] Billard V, Moulla F, Bourgain JL, Megnigbeto A, Stanski DR (1994). Haemodynamic response to induction and intubation. Anesthesiology.

[30] Cork RC, Weiss JL, Hameroff SR, Bentley J (1984). Fentanyl preloading for rapid sequence induction of anestehsia. Anesth Analg.

[31] Dahlgren N, Messeter K (1981). Treatment of stress response to Laryngoscopy and intubation with Fentanyl. Anesthesia.

